# Assessment of quality of life among parents of children with congenital heart disease using WHOQOL-BREF: a cross-sectional study from Northwest Saudi Arabia

**DOI:** 10.1186/s12955-019-1249-z

**Published:** 2019-12-16

**Authors:** Saad Khoshhal, Khaled Al-Harbi, Ibrahim Al-Mozainy, Saeed Al-Ghamdi, Adnan Aselan, Mohammad Allugmani, Sherif Salem, Dina El-Agamy, Hany Abo-Haded

**Affiliations:** 1 Pediatric Cardiology Team, Department of Pediatrics, Faculty of Medicine, Taibah University, Al-Madinah Al-Munawarah, Saudi Arabia; 2 Pediatric Cardiology Unit, Department of Pediatrics, Maternity and Children Hospital (MCH), Al-Madinah Al-Munawarah, Saudi Arabia; 3 Pediatric Cardiology Department, Madinah Cardiac Center (MCC), Al-Madinah Al-Munawarah, Saudi Arabia; 4 Department of Pediatrics, Faculty of Medicine, Menoufiya University, Menoufiya, Egypt; 5 Department of Pharmacology, Taibah University, Al-Madinah Al-Munawarah, Saudi Arabia; 6 Pediatric Cardiology Unit, Department of Pediatrics, Faculty of Medicine, Mansoura University, Mansoura, 35516 Egypt

**Keywords:** Quality of life, Parents, Congenital heart disease, WHOQOL-BREEF

## Abstract

**Background and aims:**

Health-related quality of life (HRQOL) has garnered increasing interest especially for health care providers and researchers. The study aims to evaluate the HRQOL in parents of congenital heart disease (CHD) children, and to clarify the effect of the disease severity on the outcome of the HRQOL perception. Also, to analyze the internal consistency of the Arabic version of the World Health Organization (WHO) QOL-BREEF tool in order to determine whether the tool had good validity for the target population.

**Methods:**

A cross-sectional study. The HRQOL perception was evaluated using WHOQOL-BREF questionnaire, and the internal consistency of the tool was tested using Cronbach’s alpha (α-C),

**Results:**

The study sample consisted of 200 individuals, 120 parents of CHD children, compared to 80 parents of children with minor illnesses (mean age of participating parents = 35.1 ± 9.8 years). While evaluating the HRQOL, the group of parents of children with minor illnesses had higher scores than the total group of parents of CHD children in all domains, indicating a better HRQOL.

Class-IV subgroup of parents of CHD children showed the most significant lower total score of domains between all classes (44.47 ± 12, *p* < 0.001). With respect to the internal consistency of the WHOQOL-BREF, estimation of α-C values were 0.84 points for the group of parents of CHD children, and 0.87 for the group of parents of children with minor illnesses.

**Conclusions:**

This short-term study emphasized that, HRQOL scores among parents of CHD children are compromised, and the severity of their children illness significantly affect the total score of domains in their HRQOL perception. Furthermore, the tool showed to be practical and efficient to evaluate the QOL of parents of CHD children in our population in future researches.

## Background

Congenital heart disease (CHD) is the most well-known reason of major congenital anomalies (incidence 8 for 1000 births globally), representing a major public health problem. The exact cause of CHD is often unknown but 10–20% of the defects are secondary to genetic and environmental factors [1, 2].

The advancement in the prenatal diagnostic tools have considered the early diagnosis of the congenital defects and more appropriate post-natal management. Since 1995, almost 9 out of 10 CHD child may reach adulthood [3].

Despite an increase in life expectancy, CHD may still place psychological, physical and economic burdens on children and their families. The child well-being status could be further affected by the parents’ psychosocial status. On the other hand, the parents’ physical function and psychological health could be affected by their child’s health [4, 5].

Health-related quality of life (HRQOL) is defined as “a multidimensional construct which includes physical, social, and psychological functioning, has risen as an essential result in pediatric populaces with chronic health conditions” [6]. HRQOL assessment is particularly significant in debilitating perpetual conditions that drive patients to change their ordinary routines or keeps them from completing their normal daily activities [7]. Parents of children living with a chronic illness may be overpowered by physical, social, emotional, and financial related vulnerabilities [8–10]. Evaluation of the parents’ psychological status, psychosocial status and physical functioning is important, since taking care of a child with a chronic disease will require parental support and family adaptation. Awareness and management of the family’s needs are associated with improved medical outcomes in the patient results [11].

Research studies conducted on the Arab population reporting parents’ perceptions of their own HRQOL and the stigma related to caring for a child with CHD are few, and even fewer studies were carried out in Saudi Arabia (SA) [12, 13]. These studies concluded that CHD affect all parts of HRQOL of patients and their families, and they recommended social, educational and psychological support for patients and their parents as a critical factor for making strides to HRQOL. Consequently, the essential aim of this study is to compare the HRQOL perception in parents taking care of a CHD child to parents of children with minor illnesses in Northwest region, Saudi Arabia (SA), and to show the effect of the disease severity on the outcome of the HRQOL perception in parents. Also, the secondary objective is to evaluate the internal consistency of the Arabic version of WHOQOL-BREF instrument to ensure its validity for the study group.

## Methods

### Study design and participants

A comparative cross-sectional study. Data were gathered over a period of 12 months from December 2016 to December 2017 in two large pediatric cardiology units {in Maternity & Children Hospital (MCH) and Madinah Cardiac Center (MCC)}, in Madinah district, Northwest region, SA. According to the number of populations in the Northwest region, the prevalence of children with CHD and the number of children following-up in these two units, a sample of 200 participants was considered. One-hundred-twenty parents of children with CHD, who get together with the inclusion criteria, were recruited from both centers over the study period, compared to 80 random sampled parents joining their children who were visiting the general out-patient clinics for minor diseases (e.g. upper respiratory tract infection, abscess, sore throat, and diarrhea).

The inclusion criteria were: (a) the child had been diagnosed with CHD by a pediatric cardiologist, more than 6 months preceding the beginning of the study; (b) the child age between one to ten years old, as after this age the child can self-report his perceptions; (c) the attending parent was fluent in Arabic language and had agreed to be enrolled in the study with written informed consent; and (d) lived in Northwest Saudi Arabia. The exclusion criteria included the following: (a) parents of children with CHD associated with any other extreme chronic illness (e.g. neurodevelopmental diseases or chronic kidney disease); (b) parents who had experienced a major traumatic event at least 6 months before data collection, such as a divorce or death of someone close to them, as such events might negatively impact their HRQOL, but which is not directly related to taking care of a child with CHD. In the group of parents of children with minor illnesses, parents with a second child with CHD or another child with any other chronic disease, were exclude as this might influence the study results.

Clinical data such as the type of CHD, treatment used, and number of admissions since diagnosis was obtained by the attending cardiologist and from the medical records. The Severity of heart disease was rated by a clinician who was blinded to QoL outcomes. The children with CHD were divided into four groups, contingent upon the severity classification of CHD: {class I, Mild CHD}: needing no medical treatment or effectively treated non-operatively (e.g. catheter therapy); {class II, Moderate CHD}: surgically corrected (curative) and needing no medical treatment; {class III}: surgically treated with significant residua and requiring medical therapy or in need for another surgery; {class IV, Complex or severe CHD} uncorrectable or palliated CHD (e.g. single ventricle). The CHD severity classification was adopted from a previous study by Uzark K et al. [14]. Ethical approval was taken from Taibah University Ethical Committee Review Board.

### Procedure and collecting data

Personal interviews with all participating parents were conducted in the venue of MCH and MCC to ascertain that the participating parents go with the inclusion criteria; fully understand the items of the questionnaire and assure a reliable correct response to the questionnaire; then the parents self-reported their own HRQOL.

The research tool was the World Health Organization (WHO) QOL-BREF assessment instrument [15]. The WHOQOL-BREF Instrument is a 26-item version adapted from the WHOQOL-100 assessment to provide a rapid evaluating instrument for the health-related functions in four domains of health. It has been found to be dependable and substantial for use in different cultural groups, including Arab general population [16, 17] and thus encouraged cross-cultural applicability. The Arabic version of WHOQOL-BREF was translated by the WHO-QOL group from the English version. The authors of this study obtained a permission from WHO-QOL group to use the Arabic version of this tool.

The WHOQOL-BREF instrument consists of four domains: physical health (7 items, Q 3, 4, 10, 15, 16, 17, and 18), psychological health (6 items, Q 5, 6, 7, 11, 19, and 26), social relationships (3 items, Q 20, 21, and 22), and environment (8 items, Q 8, 9, 12, 13, 14, 23, 24, and 25); and the remaining two items are concerned with the overall perception of QOL(Q1) and satisfaction with health (Q2). The physical domain items are routine activities, mobility, sleep, and pain. The psychological domain evaluates: negative musings, inspirational attitudes, self-image, self-esteem, learning capacity, mentality, focusing, memory, religion, and mental status. The social relationships domain includes: individual connections, social help, and sexual life. Environmental domain investigates items of financial assets, social services, safety, surrounding environment, transportation, chances to obtain new abilities, information, and general conditions (air pollution, noise, etc.) [16].

The WHOQOL-BREF is graded on a 5-point Likert-type scale extending from 1 (strongly agree) to 5 (strongly disagree), the mean scores of the responses to each of the subscales gave a score for the overall HRQOL. Raw scores on the four domains were transformed to a 4–20 score and then changed linearly to a 0–100-scale, where 100 is the highest and 0 is the least QOL. The higher score indicates a better quality of life.

Additional questions which documented sociodemographic data of participants (e.g. age, sex, level of education, marital status, income), and disease-related data (e.g. type, duration of CHD, drugs) were retrieved from the data base of the hospital.

### Statistical analysis

Data was tested using SPSS, version 16.0. Descriptive statistics -mean, range and standard deviation (SD) scores- were calculated to describe the sample. Unpaired T-test (Welch’s corrected) and One-way ANOVA (followed by Tukey Multiple Comparison Test as post hoc test) were utilized to compare the means for all study variables. Chi-square test (with Yates correction) was used for comparison between percentages for all the study proportions per each group and per subgroups of CHD. The statistical level of significance was set at *p* < 0.05.

The studied psychometric property was the internal consistency by Cronbach’s alpha (α-C) test, which assesses whether a tool is capable of always measuring what is to be measured in the same way, producing an average correlation between questions and responses. Thus, the α-C coefficient is calculated from the variance of the individual items and the variance of the sum between items, by verifying whether all of them use the same measuring scale. Acceptable values for α-C scores were > 0.70 and < 0.95 [18].

## Results

### The socio-demographic characteristics of the parents in the study cohort

The mean age of the participating parents in the study was 35.1 ± 9.8 years (median = 34.5 years, ranged from 24.8 to 45 years). Of these, 123 (61.5%) were mothers, 178 (89%) were married, 147 (73.5%) with prevalently elementary education, and 78 (39%) were unemployed. All the analyses between group of parents of CHD children and parents of children with minor illnesses were adjusted for age and gender. The comparisons of the overall properties of both groups are exhibited in Table 1.

**Table 1:** The Socio-demographic characteristics of participating parents in the study

Characteristics of participating parents	Parents of CHD children (*n* = 120)	Parents of children with minor illnesses (*n* = 80)	*p*-value
-Age (mean ± SD)	36.8 ± 9.4	33.4 ± 10.2	0.01642^*^
-Mothers: no. (%)	68 (56.7%)	55 (68.8%)	0.1624
-Fathers: no. (%)	52 (43.3%)	25 (31.2%)	0.0705
-Married: no. (%)	106 (88.3%)	72 (90%)	0.9156
- Education			
Elementary: no. (%)	85 (70.8%)	62 (77.5%)	0.4840
High school: no. (%)	28 (23.4%)	15 (18.7%)	0.5130
University level: no. (%)	7 (5.8%)	3 (3.8%)	0.6563
-Unemployed: no. (%)	42 (35%)	36 (45%)	0.1547
-Severity class distribution of children with CHD:			
Class I: no. (%)	54 (45%)	NA	NA
Class II: no. (%)	31 (25.8%)	NA	NA
Class III: no. (%)	23 (19 .2%)	NA	NA
Class IV: no. (%)	12 (10%)	NA	NA

^*^P <0.05 considered significant, using Unpaired T-test (Welch’s corrected) for comparison between age of parents of CHD children and age of parents of children with minor illnesses

^#^*P* <0.05 considered significant, using chi-square test (with Yates correction) for comparison between percentages in the group of parents of CHD children and group of parents of children with minor illnesses

### Clinical characteristics of CHD children

The mean age of all children participating in the study was 6.3 ± 4.8 years (median = 5.2 years, ranged from 2.4 to 9.6 years). The CHD children’s mean age was 7.8 ± 6.4 years (median = 4.8 years, ranged from 2.1 to 10.3 years), were males (57.5%) and 51 females (42.5%). The children with minor illnesses mean age was 7.4 ± 5.9 years (median = 4.5, ranged from 2.2 to 9.8 years), were male (55.3%) and females (44.7%). There was no statistical significance between both children groups for age and gender. The onset of the diagnosis of the children with CHD ranged from 6 to 14 months preceding the beginning of the study.

The parents of CHD children (*n* = 120) were divided in to 4 groups as per severity classification of CHD of their children; Class I CHD (54, 45%), Class II CHD (31, 25.8%), Class III (23, 19.2%), Class IV (12, 10%). Only 24 children (20%) received a single cardiac surgery procedure, and 78 children (65%) were on medical therapy and the remaining 18 children (15%) were neither on medication nor done surgical interventions.

### HRQOL among parents of CHD children compared to control population

**Fig. 1:**
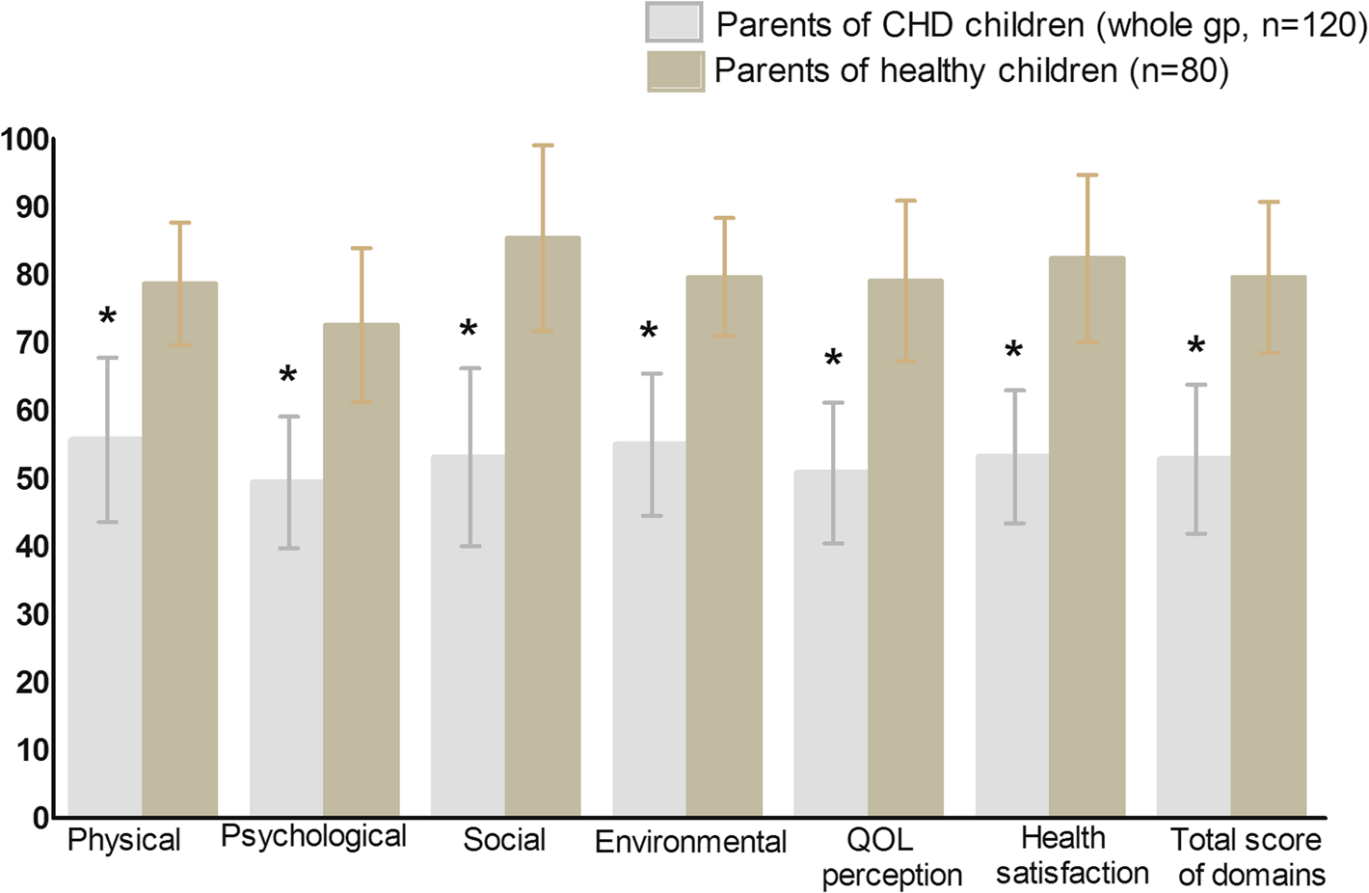
HRQOL assessment using the WHOQOL-BREF instrument

Table 2, Fig. 1 indicated that; the group of parents of children with minor illnesses (control group) had a significantly higher mean scores in all domains of the questionnaire than the total group of parents of CHD children (*p* < 0.001).

**Table 2:** Comparison of domains of HRQOL between group of parents of CHD children and group of parents of children with minor illnesses (control) using the WHOQOL-BREF questionnaire. (Data shown as mean ± SD)

Domains of the questionnaire	Parents of CHD children (*n* = 120)					Parents of children with minor illnesses (Control group) (*n* = 80)
	Class I subgroup (*n* = 54)	Class II subgroup (*n* = 31)	Class III subgroup (*n* = 23)	Class IV subgroup (*n* = 12)	Total group of parents of CHD children (*n* = 120)	
D1- Physical	66.31 ± 9.5	59.14 ± 12.3	54.49 ± 13.4	42.76 ± 13.2	55.68 ± 12.1	88.64 ± 9.02 *
D2- Psychological	62.12 ± 7.3	50.35 ± 9.8	45.87 ± 11.9	39.13 ± 9.9	49.37 ± 9.7	79.56 ± 11.3 *
D3- Social	65.4 ± 12.8	49.19 ± 11.2	52.13 ± 14.2	45.67 ± 14.3	53.1 ± 13.1	89.37 ± 13.7 *
D4- Environmental	59.89 ± 11.5	56.89 ± 9.2	55.18 ± 9.7	47.89 ± 11.5	54.96 ± 10.5	84.61 ± 8.7 *
-Perception of QOL	55.39 ± 9.2	51.74 ± 10.4	52.9 ± 7.9	43.12 ± 13.9	50.78 ± 10.4	86.05 ± 11.8 *
-Health satisfaction	54.19 ± 12.4	54.91 ± 7.8	55.29 ± 9.8	48.3 ± 9.4	53.17 ± 9.8	82.39 ± 12.3 *
Total score of domains	60.55 ± 10.5	53.7 ± 10.1 ^#^	52.64 ± 11.2 ^#^	44.47 ± 12 ^###^	52.84 ± 11	85.2 ± 11.1 *

*P <0.001 compared to total group of parents of CHD children {Unpaired T-test with Welch’s correction test was used for comparison between total group of parents of CHD children and control (group of parents of children with minor illnesses)}

^#^*P* < 0.05, ### P <0.001 compared to total score of domains of Class I subgroup {One-way ANOVA followed by Tukey Multiple Comparison Test as post hoc test was used for comparison between total score of domains of class I subgroup and other subgroups of parents of CHD children (class II, III, IV)}

In addition, there were a significant difference when comparing the total score of domains of class-I subgroup with other subgroups of parents of CHD children (class II, III, IV) (*p* < 0.05, *p* < 0.05, and *p* < 0.001, respectively). Class IV subgroup of parents of CHD children showed the most significant lower total score of domains between all classes (*p* < 0.001).

### Evaluation of the internal consistency (IC) of HRQOL-BREF questionnaire

Table 3 showed the evaluation of the internal consistency (IC) connected to groups of parents of CHD children and parents of children with minor illnesses. The α-C was applied to each item of HRQOL-BREF questionnaire, total score, and for each domain per parent group. In the investigation of each domain and the total score, the instrument acquired an estimation of α-C = 0.84 points for the group of parents of CHD children and 0.87 for the group of parents of children with minor illnesses. These values demonstrate a solid internal consistency for both groups of parents, jointly between items and in the total, as acceptable scores are between > 0.70 and < 0.95**.**

**Table 3:** Assessment of internal consistency of individual items (HRQOL-BREF questionnaire questions) using Cronbach’s alpha coefficient (α-C) applied to group of parents of CHD and group of parents of children with minor illnesses

	α-C parents of CHD children (*n* = 120)	α-C parents of children with minor illnesses (*n* = 80)
HRQOL-BREF questionnaire Items		
1. How would you rate your quality of life?	0.84	0.87
2. How satisfied are you with your health?	0.84	0.87
3. Do you feel physical pain prevents you from doing what you need to do?	0.85	0.86
4. How much do you need medical treatment to function in your daily life?	0.85	0.88
5. How much do you enjoy life?	0.84	0.87
6. To what extent do you feel your life to be meaningful?	0.84	0.87
7. How well are you able to concentrate?	0.85	0.87
8. How safe do you feel in your daily life?	0.84	0.88
9. How healthy is your physical environment (climate, noise, pollution, appeals)?	0.84	0.87
10. Do you have enough energy for everyday life?	0.85	0.86
11. Are you able to accept your physical appearance?	0.84	0.87
12. Do you have you enough money to meet your needs?	0.84	0.87
13. How available to you is information that you need in your day-to-day life?	0.85	0.87
14. Do you have the opportunity for leisure activities?	0.83	0.88
15. How well are you able to get around?	0.85	0.88
16. Are you satisfied with your sleep?	0.86	0.85
17. Are you satisfied with your ability to perform daily living activities?	0.85	0.87
18. Are you satisfied with your capacity for work?	0.84	0.88
19. Are you satisfied with yourself?	0.85	0.87
20. Are you satisfied with your personal relationships?	0.85	0.87
21. Are you satisfied with your sex life?	0.84	0.86
22. Are you satisfied with the support you get from your friends?	0.84	0.88
23. Are you satisfied with the conditions of your living place?	0.85	0.87
24. Are you satisfied with your access to health services?	0.84	0.86
25. Are you satisfied with your transportation?	0.85	0.87
26. Do you have negative feelings e.g. bad mood, anxiety, depression?	0.85	0.88
Domain		
D1- Physical (Q3,4, 10, 15, 16, 17, 18)	0.85	0.86
D2- Psychological (Q5, 6, 7, 11, 19, 26)	0.84	0.87
D3- Social (Q20, 21, 22)	0.84	0.87
D4- Environmental (Q8, 9, 12, 13, 14, 23, 24, 25)	0.84	0.87
Perception of QOL (Q1)	0.84	0.87
Health satisfaction (Q2)	0.84	0.87
Total Score	**0.84**	**0.87**

## Discussion

Congenital heart disease is considered as a standout amongst the most serious chronic disease in children [19]. Parents who are the bread winners in the family, struggle to keep up a steady proficient life, as they more often need to meet the extra requests (e.g. physical, emotional, and financial demands) of caring for a child with a chronic illness. According to some studies, parents of children with chronic diseases generally will have closer social relationships with other family members, utilized as a reason for support and procuring skills to adapt to their child’s disease [7–10]. Hopelessness, distress, and financial issues clarified more change of HRQOL than the disease seriousness. Abundance stress has been accounted more common by parents of children with heart disease in comparison with parents of healthy children [20–23].

The WHOQOL-BREF instrument was utilized in this comparative cross-sectional study. Other than CHD, the WHOQOL-BREF instrument is widely used in other sick populations -as a general measure and not disease related- such as asthma, Osteogenesis Imperfecta, cerebral palsy and children with hearing loss [7, 24–26]. In all the four domains (physical, social, psychological and environment), perception of HRQOL, health satisfaction and total score of domains assessed in the WHOQOL-BREF tool; the total group of parents of CHD children scored significantly lower values in comparison with the group of parents of children with minor illnesses (control). These findings are predictable with other studies that also found that parents of the CHD children reported significantly lower HRQOL scores compared to parents of the children with other chronic diseases or parents of children with minor illnesses [19–22].

In this study, although all domains of HRQOL were significantly affected in the total group of parents of CHD children in comparison to the control group, but the psychological domain and perception of QOL domain achieved the least scores (49.37 ± 9.7 and 50.78 ± 10.4 respectively), indicating the need of psychological support and rehabilitation in the future treatment plans. Compared to previous studies, physical well-being and psychological health were the two areas that mostly influenced the QOL of this population. These authors expressed that the physical quality is low because of sleep interference, loss of vitality and substantial grievances, impacting the physical wellbeing discernment, as well as the negative sentiments that, thus, specifically meddle with the impression of the psychological domain. Moreover, environmental components, including noise, traffic, pollution, and weather, also affect the HRQOL of parents and their children [27, 28].

The parents of CHD children (Class-IV subgroup) showed significantly lower total score of domains when compared to parents of class-I subgroup. This finding was also revealed by Areias et al. [28] who found that children -who had undergone cardiac surgery, as well as their parents- reported a lower perception on their overall HRQOL. These authors suggested that this was due to daily life confinements, post-surgery residual side effects that limit physical activity, and due to the impression of life danger and delicacy occurring in surgeries.

It is vital to utilize an evaluation tool that is psychometrically stable and is culturally diverse, to ensure that the findings are comparable across various nations. Although this study is an initial research attempts to utilize the WHOQOL-BREF instrument in our developing region for analyzing the HRQOL perception in parents of CHD children, but it was utilized several times in a wide scope of societies measuring the impact of different diseases e.g. in USA, Europe, Egypt [13, 17, 23, 29]. Furthermore, the internal consistency of the arabic version of the tool was high, with IC values of > 0.8 indicating almost perfect agreement between all items in both groups of parents. This could improve and reinforce the results of future studies, and it is possible to state that it can be applied to those subjects if its internal consistency is analyzed.

The main limitation of our study is the dependence on one parent-reported assessments for both parents. The second limitation was that the study didn’t evaluate the impact of the age of the CHD children or the disease duration on the family’s HRQOL perception. Also, the sample size was relatively small due to difficulties in attracting the participants to fill out the questionnaire. Hence, the study outcomes ought to be viewed as preliminary and long-term follow phase for these families will be required.

## Conclusions

As per our short-term outcomes and emphasizing the importance of this study, we conclude that parents of CHD children reported significantly lower scores in all domains of HRQOL compared to group of parents of children with minor illnesses. Regarding the severity of illness, the total score of domains was significantly affected in subgroup of parents of CHD children (Class-IV) compared to subgroup of parents of CHD children (Class-I). This will require a future awareness and management of the family’s needs to improve the medical outcomes of the children.

Furthermore, The Arabic version of WHOQOL-BREF instrument, showed to be practical and efficient to evaluate the QOL of parents of CHD children in our population in future researches, as long as its internal consistency is analyzed.

## Data Availability

The data that support the findings of this study are available from the Pediatric Department (Maternity and Children Hospital, Madinah, KSA), but restrictions apply to the availability of these data, which were used under license for the current study, and so are not publicly available. Data are however available from the authors upon reasonable request and with the permission of the Maternity and Children Hospital, Al-Madinah Al-Munawarah, KSA.

## Abbreviations

CHD: congenital heart disease
HRQOL: Health-related quality of life
QOL: Quality of life
WHO: World Health Organization
